# Evaluation of a Pilot Program to Increase Mental Health Care Access for Youth—The Interprofessional Child-Centered Integrated Care (ICX2) Model

**DOI:** 10.3390/children12070910

**Published:** 2025-07-10

**Authors:** Nicole Klaus, Evelyn English, Elizabeth Lewis, Jordan Camp, Sarah Krogman, Kari Harris

**Affiliations:** 1 Department of Psychiatry and Behavioral Sciences, University of Kansas School of Medicine-Wichita, Wichita, KS 67214, USA; kharris2@kumc.edu; 2 Department of Pediatrics, University of Kansas School of Medicine-Wichita, Wichita, KS 67214, USAelewis9@kumc.edu (E.L.); jcamp3@kumc.edu (J.C.); skrogman@kumc.edu (S.K.)

**Keywords:** mental health care access, pediatric mental health, integrated care, school-based health

## Abstract

Background/Objectives: The pediatric mental health crisis in the United States has reached unprecedented levels. Severe shortages in specialized health care professionals, particularly child and adolescent psychiatrists (CAPs), exacerbate the challenge of delivering timely and quality mental health care, especially in rural areas like Kansas. Innovative models such as Pediatric Mental Health Care Access (PMHCA) programs and School-Based Health Clinics (SBHCs) aim to integrate mental health expertise into primary care settings to address this gap. Methods: This paper examines an integrated care model to support SBHCs developed by the Kansas PMHCA. The Interprofessional Child-Centered Integrated Care Model (ICX2) was implemented within an SBHC in Haysville, KS. ICX2 utilizes biweekly collaborative team meetings (CTMs) via zoom involving primary care, psychology, child psychiatry, social work, and school resource coordinators to discuss patient cases and enhance the primary care management of pediatric mental health. This descriptive study analyzes data from January 2023 to June 2023, focusing on patient demographics, case characteristics discussed during CTMs, and recommendations made by the interprofessional team. Results: Findings illustrate the complex biopsychosocial needs of patients seen and define themes of case consultation and recommendations. Conclusions: Integrated care programs like ICX2 can be feasibly implemented through PMHCA programs and may be an efficient intervention to bridge resource gaps.

## 1. Introduction

The mental health crisis in the American pediatric population has progressed such that the American Academy of Pediatrics (AAP), the American Academy of Child and Adolescent Psychiatry (AACAP), and the Children’s Hospital Association have officially declared a national emergency [1,2]. As these mental and behavioral health concerns continue to rise, the number of specialized health care workers is unable to meet the unprecedented need [3,4]. The workforce, therefore, remains a critical, rate-limiting factor in providing mental health care to youths.

Kansas is no exception to this limitation, and its access is especially limited as the majority of its counties qualify as mental health professional shortage areas [5,6,7]. Extrapolating from national data, it is estimated that Kansas has nearly 50,000 students who meet criteria for severe emotional distress (SED) and/or severe mental illness (SMI) and who need school-based and specialty mental health services [8,9], yet 90% of Kansas counties have no child and adolescent psychiatrists [10]. Despite the growing need for youth mental health care in Kansas, the demand is not being met.

Pediatric Mental Health Care Access (PMHCA) programs offer one strategy for addressing workforce shortages and the maldistribution of specialists [11]. PMHCA programs aim to integrate mental health care into pediatric primary care by providing specialist consultation and educational resources to primary care physicians and clinicians (PCPs) [11,12]. PMHCA programs have been federally funded through the Health Resources and Services Administration since 2018, and there are currently 54 programs across the nation [12]. Each program has unique components based on the needs in that state, territory, or tribal nation and all provide expert consultation to PCPs, usually utilizing a child psychiatrist and/or psychologist [12].

Beginning in 2022, the HRSA called on PMHCA programs to expand into school settings to help address the growing need for mental health care amongst youths [13]. School-based services have become an important point of access for many students [14] and they complement traditional school health services with primary care, mental health care, vision, and dental care [15]. By increasing access to health care, school-based health clinics (SBHCs) have been shown to improve academic and health outcomes [16,17,18].

KSKidsMAP, the Pediatric Mental Health Care Access (PMHCA) program in Kansas, developed a model of case consultation between an SBHC and the PMHCA program’s pediatric mental health team (PMHT). This team comprises a child psychiatrist, a child psychologist, a social worker, and a pediatrician. The goal of this team is to equip the SBHC clinicians with the knowledge and skills to address the specific mental health needs of their patients. In October 2021, the Haysville Unified School District (USD 261) opened an SBHC in an underserved, low-income community. The clinic was initially staffed by an advanced practice registered nurse (APRN) and medical assistant, with a family medicine physician joining shortly after. It quickly became apparent that there was a high unmet need for pediatric mental health care in this population, with over half of visits including a mental health concern [Harris, K, unpublished program data, 2022].

Haysville is a unique population, laying outside a major metropolitan area in the rural state of Kansas. Despite resources within a 20 min drive, socioeconomic barriers make access to care difficult. Nearly 60% of the student body qualify for free or reduced lunches, attendance rates are in the bottom quintile of all Kansas districts, and dropout rates are high, with Haysville ranking in the top fifth of the state for high school incompletion [19]. Other than the SBHC, there is only one other recently opened clinic in the township. To better serve students, a part-time child and adolescent psychologist was co-located with the primary care team at the SBHC to provide psychotherapy services for patients. The SBHC primary care team utilized KSKidsMAP for guidance on screening, diagnosis, and treatment. Still, a need for further guidance on psychiatric care was identified. Thus, the SBHC team designed a pilot program to address the unmet need. The support offered by KSKidsMAP was expanded and the Interprofessional Child-Centered Integrated Care Model (ICX2) was developed.

The ICX2 pilot began in September 2022 and centers around a 90 min collaborative team meeting (CTM) held every other week with the primary care team, the psychology team, a child and adolescent psychiatrist (CAP), a social worker, and a school resource coordinator. While direct clinical care occurs solely through the primary care team and psychologist, the members of the ICX2 team provide input, consultation, and education regarding the direct management of children and adolescents with mental health concerns.

The primary goal of the model is to provide expert-informed mental health care for youth while reducing barriers and increasing access to essential health services. Given the scarcity of highly trained mental health experts such as CAPs, this model aims to use their expertise in an efficient way that also builds the capacity of PCPs to directly provide high-quality mental health care. Ultimately, the model could help to build up the currently insufficient workforce to meet the overwhelming demand for mental health care. This research study analyzes data from the ICX2 program with the aim to describe the utilization, case characteristics, and recommendations provided through ICX2.

## 2. Materials and Methods

### 2.1. Study Design

This project is a preliminary descriptive study of the ICX2 model, using a convenience sample of students who received services at the Haysville SBHC and were discussed during the ICX2 CTMs over a six-month period (18 January 2023 to 21 June 2023). Quantitative and qualitative data were abstracted from patient electronic health records in the electronic medical record and were matched with data abstracted from bi-weekly collaborative team meeting minutes. The University of Kansas School of Medicine-Wichita Institutional Review Board approved this study.

### 2.2. Sample

All unique patients between the ages of 3 and 21 years who were discussed during the ICX2 CTMs during the study period were eligible for inclusion. Patients were only excluded if the meeting minutes did not provide sufficient information for analysis.

### 2.3. Data Collection

CTM minutes were initially collected to provide written feedback to the primary care team. These CTM minutes were secondarily analyzed for study purposes. Information from the CTM minutes included the date(s) each patient was discussed, time spent discussing each case, a narrative case overview, factors considered during the case discussion (e.g., biological, psychological, social, cultural/diversity, developmental, or spiritual), specific recommendations for the case, and categories of those recommendations (e.g., psychiatric evaluation, psychopharmacological change or initiation, higher level of care referral). Clinicians who provided direct care to each patient included in the study matched CTM minutes to patient identifiers so that demographic information could be collected from the medical record. Deidentified data for each unique patient was then entered into a secure REDCap™ [20,21] database, associated with a research identification number. Patient demographics were also collected from the electronic medical record and included the following: the age at the time of the first visit to the SBHC, race, ethnicity, biological sex, gender identity, mental health diagnoses, insurance payer, medications, and dates of visits during the study period. CTM minute information was then entered for each consultation that occurred for that specific patient, as some patients were discussed in more than one CTM.

### 2.4. Analysis

Demographic data and quantitative characteristics of cases and recommendations are reported as frequencies and percentages. Qualitative analyses of case characteristics and recommendations were completed using a grounded theory approach to identify themes [22]: two medical professionals on the research team independently reviewed all of the compiled CTM minutes to identify themes and examples of the case and recommendation characteristics based on their clinical expertise, then met together with the research team to discuss and reach a consensus on broad themes and examples of each theme.

## 3. Results

### 3.1. Patient Demographics

A total of 27 patients were included in this study, with ages ranging from 5 to 18 years old, averaging 11.5 years (SD = 3.8). Patients were primarily cisgender (96%, *n* = 26/27), and slightly more than half were biologically female (56%, n = 15/27). Most patients discussed were White (89%, *n* = 24/27), 7% were Black (*n* = 2/27), and 4% were “Other” (*n* = 1/27). In terms of ethnicity, 11% (*n* = 3/27) were Hispanic/Latinx. Most patients were insured through Medicaid (78%, *n* = 21/27), with 18% covered under private insurance (*n* = 5/27) and 4% covered under TriCare (*n* = 1/27).

The majority (85%, *n* = 23/27) of patients had more than one diagnosis, and almost 50% (*n* = 13/27) had three or more diagnoses, ranging from two to five per patient for an average of 2.48 overall (SD = 0.98). The most common Diagnostic Statistical Manual Fifth Edition (DSM 5) diagnoses listed in the medical charts of included patients were major depressive disorder (MDD), generalized anxiety disorder (GAD) (52% each, *n* = 14/27), and attention-deficit hyperactivity disorder (ADHD; 48%, *n* = 13/27). Oppositional defiant disorder (ODD), social anxiety disorder, and adjustment disorder were each present in 7% (*n* = 2/27) of the study population, and post-traumatic stress disorder affected 4% (*n* = 1/27). Behavioral issues that did not meet DSM 5 criteria for a more specific diagnoses accounted for 33% (*n* = 9/27) of diagnoses. Over one-third (37%; *n* = 10/27) of patients had diagnoses categorized as “Other”. The “other” category includes both very precise diagnoses with low numbers and unclear diagnoses that did not otherwise meet DSM 5 criteria for a specific diagnosis. Examples of “other” diagnoses were trauma-related circumstances, specific substance use, and rule-out diagnoses.

### 3.2. Qualitative Analysis of Consultation Characteristics

For the 27 unique patients included in the study, there were 50 total case consultations, meaning that for some patients, clinicians consulted the team more than once. The characteristics raised during these case consultations most often concerned social factors (82%, *n* = 41/50), followed by psychological (70%, *n* = 35/50) and biological (52%, *n* = 26/50) factors. Developmental factors were considered in 14% of cases discussed, cultural/diversity factors were considered in 4% (*n* = 2/50), and spiritual factors were discussed once (2%, *n* = 1/50). The mean time spent for each case’s discussion was 12 min (SD = 8.51).

### 3.3. Qualitative Analysis of Case Characteristics

Analysis of case consultation characteristics presented to the ICX2 team found themes of (1) school-related issues, (2) social determinants of health, and (3) presenting physical/mental health concerns.

#### 3.3.1. Theme 1: School-Related Issues

School-related issues refer to problems and solutions found in the patients’ school environment, including concerns with school functioning (e.g., behavior, attendance, academic achievement) and school-based interventions (e.g., IEP, 504 plans, school behavior plans). Narrative examples from CTM minutes are listed in Table 1.

#### 3.3.2. Theme 2: Social Determinants of Health

Social determinants of health are non-medical factors that influence a patient’s mental health and wellbeing, including trauma history, grief/traumatic grief, substance use, home stressors, legal issues, sexual/gender identity, and parental mental health (Table 1).

#### 3.3.3. Theme 3: Presenting Physical and/or Mental Health Concerns

The final theme identified by the qualitative analysis was presenting physical and/or mental health concerns. These concerns included concerns related to reproductive health, developmental concerns, symptoms related to psychiatric diagnoses, behavior problems, and suicidal ideation (Table 1).

### 3.4. Qualitative Analysis of Recommendation Characteristics

Analysis of recommendations from the CTMs identified three primary categories: (1) treatment, (2) coordination of care, and (3) primary care physician/clinician education.

#### 3.4.1. Theme 1: Treatment Recommendations

Recommendations for specific treatments included medication recommendations, non-pharmacological treatments, and referrals for higher levels of care. Narrative examples from CTM minutes are listed in Table 2.

#### 3.4.2. Theme 2: Coordination of Care Recommendations

Coordination of care refers to the collaborative effort to coordinate care between the primary care team and the psychologist, including the discussion of referrals among the SBHC team, symptom improvement/progress updates, and concerns about worsening symptoms and/or safety risks (Table 2).

#### 3.4.3. Theme 3: Recommendations Related to Primary Care Physician/Clinician Education

Recommendations related to primary care physician education included screening measures, medication side effects, symptom recognition, motivational interviewing strategies, trauma-informed care, education about specific therapeutic modalities, and communication strategies for working with parents and schools (Table 2).

## 4. Discussion

High-quality mental health support for youths is needed, especially in rural states like Kansas where specialist care is extremely limited [2]. Because it takes many years to train experts like CAPs, workforce shortages will persist, and innovative models are needed to care for youths until workforce needs can be met. Interprofessional care and consultation integrated into school-based health care clinics is one method that may address this need.

Similarly to other studies looking at mental health needs in SBHCs, this study highlights that SBHCs are a major source of mental health care for youths. Often, primary care physicians and clinicians feel ill-equipped to manage mental illness in their practices [23]. This study describes a novel approach to supporting PCPs in the management of youths with mental illness by efficiently utilizing a highly trained and interprofessional team through partnership with the KSKidsMAP PMHCA program. Additionally, findings shed light on the types of patients for whom PCPs wish to consult with experts, as well as the types of recommendations interprofessional teams can provide to PCPs.

The characteristics of cases discussed in the ICX2 CTM highlight the complex, intertwined biopsychosocial needs of the patients seen in the SBHC. The majority of patients discussed had comorbid conditions, with 48% having more than three concurrent diagnoses. Additionally, many patients discussed had experienced negative social determinants of health. Most cases discussed included multiple overlapping concerns from the themes identified. As youths spend most of their time in school, physical and mental health concerns were often noted to impact and be impacted by school issues and social determinants of health. It is therefore unsurprising that as a medical care desert, the Haysville school district also struggles with student attendance and matriculation [19]. These biological, psychological, social, and academic issues should not be addressed independently from each other. Consistent with these case complexities, analysis revealed that ICX2 consultations emphasized a biopsychosocial formulation of cases allowing for discussion with the interprofessional team that included a school-resource coordinator. Qualitative analysis of recommendations reveals that multi-modal treatment recommendations and care coordination were commonly incorporated, which supported connection with community resources and collaboration between school and health care systems. Finally, the ICX2 model was able to efficiently address the educational needs of the primary care team in real time related to current, relevant cases. The educational needs identified for this SBHC team (e.g., screening measures, medication side effects, symptom recognition, motivational interviewing strategies, trauma-informed care, education about specific therapeutic modalities, and communication strategies for working with parents and schools) may inform educational efforts in other SBHCs or beyond to general primary care practices.

While this is a small pilot program with a small sample size in an under-resourced community, some aspects are likely generalizable. For instance, the average discussion time per patient consultation was 12 min, indicating that this model may be highly time-efficient. Traditional visits with a CAP and/or psychologist often include lengthy waits to establish care, followed by longer patient visit times, and require additional resources for travel to larger, metropolitan areas [23]. According to the APA’s 2023 Practitioner Pulse Survey, two-thirds of psychologist respondents reported wait times of up to three months, while the remainder of respondents reported wait times of over three months [24]. Utilizing PMHCA programs to implement expert-informed mental health care within SBHCs can allow for a reduced travel burden and quicker access not only to a medical professional, but also to the expertise offered by a highly trained specialist.

The evaluation of this novel program was impacted by a few limitations. As noted above, one limitation is that this study only focuses on themes identified in consults for 27 patients. While these themes are likely generalizable for children and adolescents in other similar communities, there may be some differences in issues faced by patients in other geographic areas. Additionally, the convenience sampling method introduces potential selection bias that can also affect the generalizability of the study results. The short timeframe of the study limits the ability to observe long-term model sustainability, and the focus on process evaluation rather than clinical outcomes limits conclusions about the model’s effectiveness in improving patient mental health. Still, despite these limitations, the study team recognized the urgency to disseminate findings that may benefit similar populations during this pediatric mental health crisis.

Further study of the ICX2 model and other SBHC–PMHCA partnerships is needed, including larger sample sizes and longitudinal data to better assess effectiveness and sustainability, as well as the quantitative analysis of patient outcomes. For example, progressive research could be directed at evaluating patient and family satisfaction, educational impact, and clinical outcomes using various psychological measures, school data (i.e., grade reports, assessments, etc.), and patient-reported surveys to assess the model’s effectiveness. Research is needed in other health care professional shortage areas to better understand the needs of populations that typically have limited access to mental health care.

Unfortunately, finding funding for a program like ICX2 may be a barrier to implementation. This study and the ICX2 CTMs were funded through the Bipartisan Safer Communities Act (BSCA) as an expansion to the Health Resources and Services Administration (HRSA) PMHCA grants allowing for compensation to the given to the ICX2 professionals during CTMs and for the research to be completed. Still, with further evaluation—especially related to patient outcomes and physician/clinician satisfaction, knowledge change, comfort gain, and practice change—insurers may become motivated to cover the costs of interprofessional consultations.

## 5. Conclusions

Shortages in highly trained pediatric mental health professionals are ubiquitous across the country, but that burden is increased for youths living in rural states like Kansas and especially for those living in health professional shortage areas. Workforce shortages will not be quickly corrected and therefore innovative methods that deliver high-quality care need to be utilized and studied while addressing the greater need for training professionals. This study contributes to the current literature by describing one such innovation that utilizes an SBHC–PMHCA partnership to assist PCPs who care for youth in an under-resourced community. These findings may inform other PMHCA programs or groups seeking to provide support and consultation for schools and primary care.

## Figures and Tables

**Table 1:** Theme examples of case characteristics.

Theme	Description	Narrative Examples from Meeting Minutes
School-Related Issues	Problems and solutions found in the patients’ school environment	Mother reports patient failed all classes, but school moved her to next grade. Patient dislikes school and doesn’t feel like she’ll do well in the upcoming year. Truancy has been an issue. Discussed increasing medication to improve school behavior. Teachers report him doing better in class, but still distracts/irritates other students and shows impulsive behaviors. Currently suspended for vaping at school. Trouble focusing in school, social anxiety, struggles talking with/in front of people (does better in small groups).
Social Determinants of Health	Non-medical factors that influence patient health and wellbeing	Lived with grandparents due to mother’s substance abuse. Patient has been more gender-neutral in behavior and expressions. They don’t know their gender or sexual identity. Most of their friends are LGBT+. Mom is very religious; patient has been doing faith-based therapy where they don’t feel able to open up about these issues. Molested as a young child by family relative. Self-medicates through marijuana use to manage anxiety and depression. Trauma history, unstable home life, severe injuries as a young child. Caught shoplifting and did intake at juvenile detention center. Housing instability, moved in with family friends and bounced back and forth between homes resulting in child being out of school for a couple months. Dad died of suicide after patient moved to current home, guardian has concerns about how patient is dealing with grief. Mom expressed she’s overwhelmed as essentially a single parent with low funds, foregoing her own therapy to support her children—parent mental health is likely taking a toll on this situation.
Presenting Physical and/or Mental Health Concerns	Patient’s mental or physical health concerns	Patient reported suicidal ideation. Later denied being suicidal, but admitted to self-harm via cutting arm. High symptom severity rated on PHQ9 and GAD7 [mental health screening measures]. No firm diagnoses, potential MDD, GAD, possible ODD. No medication at this time; previously failed fluoxetine and sertraline through non-compliance. Offered patient birth control resources; does not seem interested in birth control. Currently living with a boyfriend. Discussed plan for pregnancy, patient expressed not feeling ready to have children, but still disappointed by negative pregnancy test. Struggling with social skills, diagnosed with ADHD, potential ASD. PCP looking to rule out or potentially diagnose ADHD. Hyperactive and inattentive issues, hard to sit down during circle time, self-soothes by rubbing fingers. Not defiant or aggressive, pesters his brother but not outside of normal. Teacher Vanderbilt indicated some aggression (causing fights, teasing others). Student seems tired all of the time, trouble retaining information. Meth exposure in utero. Patient diagnosed with ADHD inattentive, currently takes Quillivant [methylphenidate] 6 mL (30 mg 1× daily). PCP concerned about decrease in weight; no weight gain in 8 months. Good symptom control, doing well in school.

**Table 2:** Theme examples of recommendation characteristics.

Theme	Description	Narrative Examples from Meeting Minutes
Treatment Recommendations	Recommendations for specific treatments	Recommend using stimulant rather than SSRI. Current medication dose may be too high for patient’s weight—PCP should monitor closely. For now, continue current course of action under close supervision. Patient should be evaluated for IEP despite absences, recommend parent call local Parent Training and Information (PTI) Center for support in advocating for patient. Recommend DCF report, family preservation resources, and adolescent substance use treatment. Patient may need further medical workup, referral to endocrinology. Start family supports and behavioral interventions, look into developmental history. Recommend trauma-focused cognitive behavioral therapy (TF-CBT). Refer to CAP.
Coordination of Care Recommendations	Collaborative effort to coordinate care between the primary care team and psychologist	Work with school to create positive behavior chart, psychologist will coordinate with PCP to write a letter to school. Continue with therapy, check on waiting list for neuropsychological evaluation. PCP will contact patient about reengaging in therapy. Suggested more evaluation (ADOS).
Recommendations related to Primary Care Physician/Clinician Education	Recommendations for PCPs to enhance their understanding and education.	Discussion of laws and when it may be appropriate to report parent behavior. Education about PHQ9 scoring, interpretation, and follow-up. Education on use of motivational interviewing to reduce substance use and build motivation to attend therapy. Discussed types of psychotherapy and how to assess whether patient is receiving evidence-based therapy for their diagnosis. Education about ways ADHD symptoms affect functioning beyond academics, the risks of untreated ADHD, and the impact of parenting styles on child behavior. Education about normal development and how to validate and educate parents. Recommendations about how to properly titrate medicine according to weight.

## Data Availability

The original contributions presented in this study are included in the article. Further inquiries can be directed to the corresponding author(s).

## Abbreviations

The following abbreviations are used in this manuscript:

CAP	Child and adolescent psychiatrist
ICX2	Interprofessional Child-Centered Integrated Care Model
PMHCA	Pediatric Mental Health Care Access Programs
SBHC	School-Based Health Clinic
PCP	Primary Care Physician/clinician
CTM	Collaborative Team Meeting
SED	Severe emotional distress
SMI	Severe mental illness
APRN	Advanced practice registered nurse
PCP	Primary Care Provider
DSM 5	Diagnostic Statistical Manual Fifth Edition
MDD	Major Depressive Disorder
GAD	Generalized Anxiety Disorder
ADHD	Attention Deficit Hyperactivity Disorder
ODD	Oppositional Defiant Disorder
IEP	Individualized Education Plan
ASD	Autism Spectrum Disorder
PHQ9	Patient Health Questionnaire screening measure
GAD7	General Anxiety Disorder screening measure
SSRI	Selective Serotonin Reuptake Inhibitor
PTI	Parent Training and Information Center
DCF	State Department of Children and Families
TF-CBT	trauma-focused cognitive behavioral therapy
BSCA	Bipartisan Safer Communities Act
APA	American Psychological Association
